# Early interventions for self-harm among children and young people in educational and primary-care settings: a scoping review

**DOI:** 10.1080/28324765.2026.2683019

**Published:** 2026-07-21

**Authors:** Andrew Sweetmore, Heidi Singleton, Ann Luce, Debbie Holley, Morad Margoum

**Affiliations:** a University of Bournemouth, Bournemouth; b University of West of Scotland, Paisley; c HealthCare University NHS Foundation Trust, Dorset

**Keywords:** Scoping review, young people, self-harm, schools, general practice

## Abstract

Self-harm among children and young people is a major public-health concern associated with substantially elevated risk of adverse outcomes including suicide. Educational and primary-care settings are frequent points of contact, prompting diverse prevention and early-intervention efforts, yet the scope and effectiveness of these remain unclear. This scoping review mapped and synthesised empirical studies evaluating interventions designed to prevent or reduce self-harm in individuals aged 25 years or younger within educational or general-practice contexts. Following JBI and PRISMA-ScR guidance, seven databases and grey-literature sources were searched to July 2025. Twenty-two studies met inclusion criteria: five randomised controlled trials, five other quantitative evaluations, four mixed-methods studies, seven qualitative enquiries, and one implementation report. Most were delivered in educational settings; primary care remained under-represented. Psycho-educational programmes were most common, followed by screening or early-identification approaches, mindfulness or expressive-therapeutic interventions, an integrated service model, professional training, and a digital self-help app. Of twelve studies with quantitative outcomes, four reported significant reductions in self-harm thoughts or behaviours, although most were small-scale and short-term. Adverse-event reporting and harm monitoring were limited. Evidence indicates potential benefits of psycho-educational and brief psychotherapeutic interventions in schools, but further multi-site trials with longer follow-up and robust harm-monitoring protocols are needed, particularly within primary care.

## Introduction

The global incidence of self-harm amongst children and young people has increased substantially over recent decades (Lim et al., 2019; Tan et al., 2025), with systematic reviews reporting lifetime prevalence estimates ranging from 16% to 22.1% in community samples of adolescents (Gillies et al., 2018; Lim et al., 2019). Self-harm refers to any act in which an individual intentionally harms themselves through poisoning or injury, regardless of whether suicidal intent is present, absent, or unclear (Hawton et al., 2003; Kapur et al., 2013; NICE, 2022). The term therefore captures the full range of self-harming behaviours, from those carried out with the intention of ending life through to those undertaken for other reasons, as well as acts where motivation cannot be reliably determined (Ougrin & Kaess, 2025).

Self-harm is associated with substantially elevated risk of adverse outcomes. A meta-analysis of 172 longitudinal studies found that prior self-injurious thoughts and behaviours predicted future suicide attempts (weighted mean OR 2.14, 95% CI 2.00–2.30) and suicide death (OR 1.54, 95% CI 1.39–1.71), though diagnostic accuracy was only marginally above chance (Ribeiro et al., 2016). Self-harm necessitating hospital presentation is associated with a 12-month suicide incidence more than 30 times higher than the expected rate in the general population (Hawton et al., 2020). Self-harm is therefore recognised as one of the strongest modifiable risk factors for suicide in young people, though the relationship is probabilistic rather than deterministic: the majority of adolescents who self-harm do not go on to attempt suicide (Mars et al., 2019). Beyond mortality risk, adolescent self-harm predicts increased risk of psychiatric morbidity, impaired social functioning, and disrupted educational trajectories (Mars et al., 2014). Furthermore, suicide is among the leading causes of death in adolescents and young adults worldwide (World Health Organization, 2021), and prevalence rates of suicidal behaviour and non-suicidal self-injury among children are substantial (Liu et al., 2022). Consequently, self-harm emerges as a pressing public-health concern, underscoring the urgency of early detection and intervention (Harris et al., 2022).

In the context of mental health care, 'early intervention' refers to the timely identification and provision of support and treatment services to children and young people who are either at risk of, or have already begun to engage in, self-harm behaviour (Ougrin et al., 2012). This encompasses both primary prevention, targeting those at elevated risk before onset, and secondary prevention, seeking to prevent escalation, recurrence, or transition to more severe outcomes among those who have already self-harmed (Dobias et al., 2023). The rationale for early intervention is threefold: epidemiological evidence demonstrates that early-onset self-harm predicts poorer long-term outcomes, including persistent self-harm, psychiatric morbidity and elevated suicide risk (Mars et al., 2014; Wilkinson et al., 2018); the majority of young people who self-harm do not access specialist mental health services (McManus et al., 2019); and early intervention may alleviate pressure on overstretched acute and specialist services by addressing difficulties before they reach crisis threshold (Edbrooke-Childs & Deighton, 2020). Recognising these imperatives, the UK Government has committed to expanding Mental Health Support Teams (MHSTs) within schools and colleges (Department for Education & Department of Health & Social Care, 2018; NHS England, 2023) whilst the National Institute for Health and Care Excellence (2022) underlines the critical role of primary care in treating self-harm and preventing recurrence.

Despite the increased prevalence of self-harm amongst young people only a small proportion will actively seek assistance, and they typically do not turn to healthcare providers for help (McManus et al., 2019). Common sources of support for young people include friends, family, teachers and general practitioners (GPs) (Rowe et al., 2014). Accordingly, it has been viewed as reasonable to orient early-intervention strategies towards essential yet non-traditional domains for self-harm treatment, such as schools (Nawaz et al., 2024), particularly considering the associated risk factors and the absence of established effective treatments for children and young people who self-harm (Harris et al., 2022).

Educational establishments (schools, colleges and universities) and primary-care services constitute pivotal access points for self-harm prevention and early intervention (Nawaz et al., 2024). Personnel within these settings, such as teachers, school nurses, pastoral staff and general practitioners, are frequently the first to notice warning signs and can provide direct support or expedite referral to specialist care pathways (Mughal et al., 2019; Nawaz et al., 2024). Yet despite growing policy emphasis on these settings, no consensus or standardised approach to early intervention for self-harm has been established across educational or primary care contexts (Department for Education & Department of Health & Social Care, 2018; Nawaz et al., 2024).

Previous systematic reviews have tended to appraise effectiveness within either educational or primary-care contexts in isolation, overlooking the inter-dependence of these settings within a wider early-intervention system (Breet et al., 2021; Thangada & Kasoju, 2024). Subsequently, to date, no review has yet mapped the full range of intervention approaches deployed across schools, colleges, universities, and primary-care settings simultaneously, nor examined how evidence from each context might inform the other.

A further limitation of the existing evidence base concerns the temporal scope of evaluation. Pilot studies conducted in educational establishments indicate the potential effectiveness of certain interventions, typically psycho-education around emotions and self-harm (Nawaz et al., 2024); however, most evaluations have been short-term in design, and there is a notable absence of evidence addressing the long-term effectiveness of these interventions in reducing the frequency and severity of self-harm among children and adolescents. Systematic reviews that have utilised meta-analyse are therefore limited not only by the siloed setting focus of the trials they synthesise, but by the short follow-up periods those trials employ.

A scoping-review approach is therefore warranted: unlike a systematic review of effectiveness, it prioritises breadth of evidence mapping over effect estimation, enabling a comprehensive account of intervention types, settings, and methodological characteristics across both educational and primary-care contexts, and making visible the evidence gaps, including those concerning long-term outcomes, that should inform future trial design (Peters et al., 2020).

## Objectives and research questions

This review aimed to chart the characteristics, thematic foci and reported impacts of empirical studies evaluating interventions for self-harm among children and young people (CYP) aged ≤ 25 years across educational and primary-care contexts, with the aim to draw out implications for practice and research.

The scoping review is structured around two research questions:

1. What early interventions do primary care services and educational institutions in high-income countries, as defined by World Bank (2024), use to support children and young people, aged 4 to 25, who engage in self-harm?
2. How effective are these interventions in decreasing the frequency and intensity of self-harm, and/or in preventing subsequent self-harm through different methods?

Both research questions are restricted to high-income countries as classified by the World Bank (2024). This restriction reflects the review's focus on settings with broadly comparable healthcare infrastructure and educational systems; the methodological rationale is elaborated in the Methods section. It is acknowledged that this boundary excludes potentially relevant evidence from low- and middle-income contexts.

## Methods

### Design and reporting

A scoping review was conducted, guided by JBI methodological guidance for scoping reviews (Peters et al., 2020) and reported using the PRISMA-ScR checklist (Tricco et al., 2018). Please see the accompanying PRISMA-ScR checklist in supplementary material for complete reporting standards compliance. This approach differs from a systematic review of effectiveness by prioritising breadth of evidence mapping over effect estimation and by not undertaking a formal risk-of-bias appraisal.

### Eligibility criteria

- **Population:** CYP aged ≤ 25 years, and/or professionals (e.g. teachers, GPs) working with them.
- **Concept:** Any intervention, programme, service model or tool explicitly aiming to prevent, reduce or manage self-harm (including ideation).
- **Context:** Educational (schools, colleges, universities) or primary-care/general-practice settings in high-income countries, as classified by the World Bank (2024).
- **Sources of evidence:** Empirical studies of any design (quantitative, qualitative, mixed-methods), including grey literature.
- **Limits:** Only studies in English included.

The exclusion of studies focused solely on suicide prevention, without explicit reference to self-harm, warrants clarification given that the review adopts an intent-agnostic definition of self-harm that encompasses suicidal behaviour (NICE, 2022). Studies framed exclusively as suicide prevention, such as those addressing lethal means restriction, typically target a different point on the clinical pathway, operate within distinct theoretical frameworks, and may be evaluated against mortality or suicidal-attempt outcomes rather than self-harm behaviour reduction or management. The inclusion of such studies would have substantially broadened the scope beyond early intervention for self-harm as a behavioural and clinical phenomenon, and would have risked conflating intervention targets in ways that obscure rather than illuminate the evidence base. Studies that addressed suicide as one component of a broader self-harm intervention were retained.

The restriction to high-income countries, as classified by the World Bank (2024), reflects the review's focus on settings with broadly comparable healthcare infrastructure, educational systems, and mental health policy frameworks. High-income countries typically share features relevant to early-intervention delivery, including universal or near-universal education systems, publicly funded or insurance-based primary care (Organisation for Economic Co-operation & Development, 2023), and national clinical guidelines for self-harm management (e.g. NICE, 2022). These structural similarities permit meaningful cross-study comparison of intervention approaches and implementation challenges. The incidence of self-harm appears to have risen most notably in high-income regions (Tan et al., 2025), further supporting the relevance of this focus. However, this restriction excludes potentially valuable evidence from low- and middle-income contexts, where self-harm prevalence is also substantial (Lim et al., 2019), and this is acknowledged as a limitation. Table 1 summarises the eligibility criteria applied.

**Table 1.** Eligibility criteria.

	Inclusion criteria	Exclusion criteria
**Population**	CYP aged ≤ 25 years; professionals (e.g. teachers, GPs) working with CYP	Adults > 25 not working with CYP; parents/carers unless part of CYP-focused early-intervention
**Concept**	Any intervention/programme/service model/tool explicitly aimed at preventing, reducing, or managing self-harm (including ideation)	Studies focusing solely on suicide prevention without mention of self-harm
**Context**	Educational (schools, colleges, universities) or primary-care/general-practice settings	Specialist mental-health services, inpatient settings
**Source of evidence**	Empirical studies (quantitative, qualitative, mixed-methods), grey literature	Editorials, opinion pieces.
**Language**	English	Non-English
**Date**	Up to July 2025	–

### Information sources and search strategy

We searched MEDLINE, Embase, PsycINFO, ERIC, CINAHL, Web of Science and Scopus from inception to 25 July 2025, supplemented by grey-literature sources and organisational websites. Search terms for self-harm combined Overdos* OR Self-injur* OR Self mutilat* OR Nonsuicidal self-injur* OR Self-harm* OR self-mutilat* OR ligatu* OR Suicid* with setting-specific keywords (school*, college*, university*, primary care, general practice) and relevant intervention filters. Reference lists of included studies and relevant reviews were also screened. The search yielded 2,964 records. Following removal of 2,740 duplicates in EndNote, 224 unique records were taken forward to title and abstract screening using Covidence software.

The selection of search terms reflected the intent-agnostic definition of self-harm adopted in this review (NICE, 2022). The breadth of terms was designed to capture the full spectrum of self-harm presentations: 'Self-harm*' served as the principal descriptor; 'Self-injur*' and 'Nonsuicidal self-injur*' ensured capture of studies using NSSI terminology; 'Suicid*' truncated to include suicidal behaviour, suicidal ideation, and suicide attempt; and 'Overdos*', 'ligatu*', and 'self-mutilat*' captured studies describing specific methods of self-harm. This inclusive strategy ensured that studies were not excluded on the basis of how intent was classified or which terminological framework was adopted.

#### Selection process

**Figure 1.**
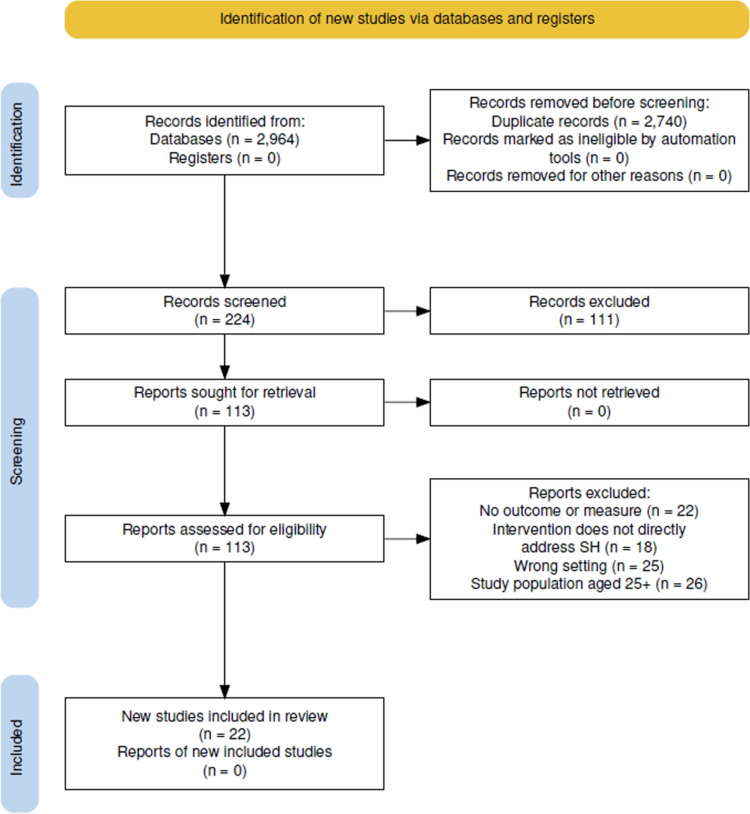
PRISMA-ScR flow diagram illustrating the study identification, screening, and selection process (Haddaway et al., 2022).

Two reviewers independently screened titles and abstracts, then assessed full texts against PCC eligibility criteria, resolving disagreements through discussion. Of the 113 full texts assessed, 22 studies met inclusion criteria. Figure 1 presents the selection process in PRISMA-ScR format (Haddaway et al., 2022).

### Data charting process and synthesis

We piloted and then used a standardised charting form to extract bibliographic details, country, setting, design, sample characteristics, intervention description (including delivery mode), outcome measures and principal findings, plus implementation issues (feasibility, acceptability, barriers/facilitators). Quantitative outcomes were summarised narratively; qualitative and mixed-methods findings were synthesised thematically. No meta-analysis or formal quality appraisal was undertaken.

## Results

### Study characteristics

The 22 eligible studies comprised five randomised controlled trials (RCTs), five other quantitative evaluations (one cohort and four pre-/post or cross-sectional designs), four mixed-methods studies (including one non-randomised controlled pilot with embedded qualitative interviews), seven qualitative enquiries and one grey-literature implementation report. Settings included schools (*n* = 10), universities (*n* = 5), other educational institutions (*n* = 1), and primary care or general practice (*n* = 6). Sample sizes (total enroled per study, where reported) ranged from 10 to 2,184 participants (median = 97 across the 19 studies reporting a quantifiable *N*). Follow-up periods varied widely, ranging from immediate post-intervention assessment only to 52 weeks. Short follow-up (under 12 weeks) was the dominant pattern; only a minority of studies (Bennewith et al., 2002; Bjärehed et al., 2013; Hart et al., 2020) extended to 26–52 weeks.

Intervention types included psycho-educational programmes, mindfulness or expressive-therapeutic small-group interventions, integrated mental-health service models, screening or early-identification tools, and professional resources or digital tools. Psycho-educational interventions typically consisted of whole-class lessons, peer-education, or clinician-authored toolkits; brief psychotherapeutic programmes were delivered in small groups to pupils or university students; integrated models included GP–Child and Adolescent Mental Health Team (CAMHS) liaison or embedded clinicians; screening tools comprised classroom or campus-based risk-identification instruments; and professional/digital resources included guideline roll-outs, workshops, and smartphone applications. A detailed summary of the characteristics and findings of all included studies is presented in Table 2.

**Table 2.** Study characteristics.

Authors (year)	Country	Study design	Age group	Sample size	Gender	Intervention type	Delivery method	Setting	Outcomes	Key findings
Bennewith et al. (2002)	UK	Cluster Randomised Controlled Trial	Adults (16 + ; mean 32.3 yrs, range 16–95)	1932	Mixed	GP invitation letter + management guidelines for GPs	In-Person	General Practice	Self-Harm Frequency	No overall effect on repeat self-harm (OR 1.17, 95% CI 0.94–1.47) iatrogenic effect in first-time presenters (interaction *p* = 0.017).
Vernberg et al. (2004)	USA	Single-group pre–post evaluation (programme evaluation)	Children (5–13 years; mean 9.6)	50	Mixed (42 boys, 8 girls; 84% male)	Integrated school mental-health service	In-Person	School	Self-Harm severity (CAFAS subscale)	Significant decrease on CAFAS Self-Harmful Behaviours subscale (M 11.0 → 2.2; t(49) = –4.49, *p* < .001; d = 1.05); 84% showed clinically significant improvement on CAFAS total.
Muehlenkamp et al. (2010)	USA	Single-arm pre–post	High-school adolescents (mean 16.07, SD 1.32)	274	Mixed	Psychoeducation	In-Person	Educational Institution	Self-Harm Frequency	No iatrogenic effects; non-significant trend toward NSSI decrease (*p* < .08); significant improvements in knowledge and help-seeking attitudes
Bjärehed et al. (2013)	Sweden	Mixed-Methods Feasibility Study	Early adolescents (Grade 7–8; mean 13.7–14.7 yrs)	97 interviewed (66 self-injury + 31 non-injuring) from survey of 1,052	Mixed	Screening + validating interview	In Person	School	Acceptability, attractiveness & iatrogenic effects of screening + interview	Adolescents reported positive feelings about interview; no iatrogenic effects at 1-year follow-up.
Birrane et al. (2015)	Ireland	Mixed Methods Feasibility Study	Adults	30 attended (24 completed evaluation)	Not Specified	Educational training workshop	In Person	General Practice	Knowledge gain & Usefulness	Positive.
Cheng et al. (2010)	USA	Cross-sectional screening-tool development	Adults (mean 22.6 yrs, range 18–62)	2,184 (1,048 women; 1,136 men)	Mixed	Development of screening tool for Nonsuicidal Self-Injury	*Online self-report mental-health survey*	University	Development and validation of a screening inventory	Screening feasible for early identification but not diagnostic.
Fukumori et al. (2017)	Japan	Randomised Controlled Trial (RCT)	Young Adults (18–22 yrs; mean 19.1–19.4)	22 (10 intervention + 12 control)	Mixed	Therapeutic Structured Writing.	In-Person	Uni	Self-Harm Thoughts	Significant short-term effects on emotion regulation and acceptance; limited effect on self-harm ideations
Bailey et al. (2019)	UK	Mixed-Methods Study	Young people GPs, practice nurses and young people (focus groups)	285 patient records + 45 focus group participants	Mixed	Psychoeducation Materials	In-Person	General Practice	Feasibility of adopting self-help assisted interventions in GP surgeries.	Positive re materials, ambivalent about use.
Lloyd-Richardson et al. (2020)	United States, Canada, Australia, Belgium	Practice framework/qualitative case analysis	Youth/adolescents (target population)	N/A	Mixed	Brief assessment and referral framework for NSSI	In-Person	School	Guidance for school nurses in screening, support, and referral	Practice guidance; SOARS framework described—no empirical effectiveness evaluation
Roberts et al. (2019a)	UK	Cohort Study	Adolescents (13–17 years)	622 programme participants (299 with pre-/post-data); 8,440 screened	Mixed	Mind and Body group programme (8 weekly group sessions + 3 one-to-one)	In-Person	School	Self-Harm Frequency & thoughts	Significant reduction in self-harm thoughts (67%) and acts (64%); improvements in mental well-being
Roberts et al. (2019b)	USA	Qualitative Study	Adults	12	Mixed	Counselling and psychoeducation	In-Person	School	counsellors’ experiences, roles, interventions, and referral practices	School counsellors used supportive, educational, and referral-based approaches to manage adolescent self-harm but reported uncertainty about their role and a need for more training.
Baetens et al. (2020)	Belgium	Non-randomised mixed-methods pilot study	Early adolescents (11–15 yrs; mean 12.85)	651 (311 Happyles + 340 HappylesPLUS)	Mixed	Psychoeducation	In-person (with online components)	School	Self-Harm Frequency	No iatrogenic effects; reduced likelihood of future NSSI engagement; increased emotional awareness
Bellairs-walsh et al. (2020)	Australia	Qualitative Study	Young people aged 16–24	10	Mixed	Qualitative focus groups on primary-care practices.	In-Person	General Practice	Youth perspectives on GP identification, assessment, and care	YP preferred collaborative, empathetic, and individualised GP approaches over risk-labelling or checklist assessments
Hart et al. (2020)	Australia	Cluster randomised crossover trial	Adolescents (15–17 yrs; mean 15.87)	1,605	Mixed	Psychoeducation	In-Person	School	Recognition of suicidality & intentions to help a suicidal peer	Training improved recognition of suicidality and intentions to help a suicidal peer (vignette-based). No evidence of greater psychological distress at 12 months on the Kessler Psychological Distress Scale, supporting absence of iatrogenic effect.
Argento et al. (2022)	Canada	Randomised Controlled Trial (RCT)	Young Adults (≥18 years; mean 20.17, SD 1.98)	144	Female	Brief mindfulness intervention	In Person & Online	University	Mindfulness increase and stress reduction	Intervention increased mindfulness and reduced stress equally among university students with and without a history of self-injury.
Cipriano et al. (2022)	Italy	Pilot uncontrolled pre–post feasibility study	Pre-adolescents (11–13 yrs; mean 11.59)—6th/7th grade	58	Mixed	Peer education/psychoeducation (school-based)	Online/Digital	School	Risk factors: emotion regulation, self-esteem, body image, alienation, maturity fear	Significant improvements in emotion regulation, self-esteem, body image, alienation, maturity fear. (Students reporting current NSSI excluded.)
Townsend et al. (2022)	Australia	Qualitative	Adults (school psychologists; discussing children < 13)	17	Mixed; 78% female	School-based management and response strategies for self-harm	In-Person	School	Qualitative	Participants stated need for training to improve early intervention.
Cliffe and Stallard (2023)	UK	Qualitative	Young Adults (18–31 yrs; mean 20.6)	25	Mixed	Qualitative study.	Online (Microsoft Teams)	University	Preferences for and experiences of support	Students preferred varied (often digital) support, and recommended outcomes focusing on wellbeing and coping.
Cliffe et al. (2023)	UK	Qualitative	Young Adults (18–31 yrs; mean 20.6)	25	Mixed	Digital self-help app (BlueIce).	Online	University	Acceptability of app.	University students found the app acceptable for managing self-harm, valuing its accessibility, though recommending it as an adjunct to professional support.
Mughal et al. (2023)	UK	practice summary based on updated NICE guideline (NG225)	n/a	n/a	n/a	Summary of NICE NG225 recommendations for primary care (assessment, referral, safety planning, follow-up)	n/a	General Practice	Implementation of NICE guidance	Practice summary of new NICE NG225 recommendations for managing self-harm in primary care.
Zare et al. (2023)	Iran	Randomised Controlled Trial (RCT)	Adolescents (15–17 yrs; mean 15.95)	191 (99 intervention + 92 controls)	Female	Psychoeducation	In-Person	School	Self-Harm Frequency	Decreased self-harm behaviours
Mughal et al. (2024)	UK	Qualitative Study	Adults	15	Mixed	GP management and care strategies for young people after self-harm	Online & telephone	General Practice	Strategies used.	GPs used standard assessment, safety planning and proactive follow-up.

### Research question 1: what early interventions do primary care services and educational institutions in high-income countries use to support children and young people (aged 4–25) who engage in self-harm?

Several broad approaches were identified across the 22 studies: psycho-educational programmes (*n* = 8), mindfulness or expressive-therapeutic interventions (*n* = 2), integrated service-link models (*n* = 1), screening or early-identification approaches (*n* = 3), professional guidance/training (*n* = 1), digital self-help/app (*n* = 1), and other or contextual studies (*n* = 6).

Interventions were most frequently deployed in schools, followed by universities, other educational settings, and primary care. Delivery modes varied. Universal classroom psycho-education was used in one study (Baetens et al., 2020), online peer-led psycho-education in another (Cipriano et al., 2022), and a targeted small-group programme following population screening in a third (Roberts et al., 2019a). Other modalities included a brief mindfulness intervention (Argento et al., 2022), an individual structured-writing exercise (Fukumori et al., 2017), an integrated school mental-health service (Vernberg et al., 2004), screening or early-identification approaches (Bjärehed et al., 2013; Cheng et al., 2010; Lloyd-Richardson et al., 2020), and staff training or digital tools to enhance recognition and response (Birrane 2015; Cliffe et al., 2023; Hart et al., 2020).

An emerging digital strand was observed in three studies; a school-based eHealth/online prevention programme, a university app study, and a hybrid psycho-educational RCT (Baetens et al., 2020; Cipriano et al., 2022; Cliffe et al., 2023).

### Research question 2: how effective are these interventions in decreasing the frequency and intensity of self-harm, and/or in preventing subsequent self-harm through different methods?

Among the 12 studies reporting quantitative outcomes—five RCTs (Argento et al., 2022; Bennewith et al., 2002; Fukumori et al., 2017; Hart et al., 2020; Zare et al., 2023) and seven other quantitative or mixed-methods evaluations (Baetens et al., 2020; Bailey et al., 2019; Bjärehed et al., 2013; Cipriano et al., 2022; Muehlenkamp et al., 2010; Roberts et al., 2019a; Vernberg et al., 2004)—4/12 reported statistically significant reductions in self-harm-related outcomes following intervention, though the outcome measures and study designs varied. Roberts et al. (2019a) demonstrated significant reductions in directly measured self-harm thoughts and behaviours in an adolescent sample; Zare et al. (2023) demonstrated significant reductions in NSSI behavioural intention and related social-cognitive constructs in female adolescents; Baetens et al. (2020) reported a reduced likelihood of future NSSI engagement from pre- to post-intervention within both study arms; and Vernberg et al. (2004) reported a decrease on the CAFAS Self-Harmful Behaviour subscale, a clinician-rated functioning measure. Effect sizes ranged from small to large and were most evident in school populations receiving universal psycho-education or a targeted school-based programme.

By contrast, 8/12 studies reported null or mixed findings, with some outcomes showing no significant change despite improvements in other measures (Bailey et al., 2019; Bennewith et al., 2002; Fukumori et al., 2017).

Durability of effects remains uncertain: most trials had small sample sizes (*n* < 200) and had short follow-up (<12 weeks), with only a minority extending to 26–52 weeks (Bennewith et al., 2002; Bjärehed et al., 2013; Hart et al., 2020). Studies of integrated service-link models and screening tools more often demonstrated improvements in identification, referral and access than direct reductions in self-harm outcomes (Bailey et al., 2019; Bennewith et al., 2002; Cheng et al., 2010; Mughal et al., 2023; Vernberg et al., 2004). Professional or digital resources, whether directed at staff (e.g. training and capacity-building: Birrane et al., 2015; Hart et al., 2020) or at children and young people (e.g. app-based psycho-educational or skills programmes: Cipriano et al., 2022; Cliffe et al., 2023), tended to improve engagement, confidence, or perceived competence, though few reported measurable service user-level changes.

Qualitative and mixed-methods findings emphasised high acceptability for brief, skills-focused group interventions, particularly when co-designed with students (Bjärehed et al., 2013; Cliffe & Stallard, 2023; Roberts et al., 2019b). In addition, clear referral pathways and attention to resource constraints and role boundaries were highlighted as essential (Lloyd-Richardson et al., 2020; Roberts et al., 2019b; Townsend et al., 2022). Adverse-event reporting was sparse across the included studies. Four studies built formal adverse-event monitoring into the study design (Baetens et al., 2020; Bjärehed et al., 2013; Hart et al., 2020; Muehlenkamp et al., 2010); a further four noted the possibility of adverse effects or operated a safety protocol without systematic measurement (Argento et al., 2022; Fukumori et al., 2017; Mughal et al., 2024; Roberts et al., 2019a); and the remaining 14 made no reference to adverse-event monitoring of any kind.

Overall, the evidence from this scoping review suggests that psycho-educational and brief psychotherapeutic group interventions show preliminary effectiveness in educational settings. However, the current evidence identified by this review is insufficient to determine whether these benefits are sustained beyond three months or to evaluate the effectiveness of primary care-led interventions. Caution over implementation of any intervention is therefore warranted, given the limited and inconsistent monitoring for potential iatrogenic effects. Where adverse effects were noted in the included studies, these typically took the form of transient distress during or immediately after sessions. While these effects were usually short-lived, their occurrence highlights the need for systematic harm-monitoring protocols in future trials. Without such safeguards, interventions risk inadvertently exacerbating distress among vulnerable young people, undermining both their safety and the credibility of school- or primary-care-based programmes.

## Discussion

This review synthesised evidence from 22 empirical studies spanning educational and primary-care settings, mapping the range of early-intervention approaches for self-harm among children and young people. Most interventions evaluated in the included studies were delivered in educational settings, with far fewer examples from primary care. This distribution likely reflects a research bias rather than the true pattern of help-seeking or the main locus of intervention. While most children and young people who self-harm do not seek professional help (McManus et al., 2019), those who do are likely to turn to a general practitioner or a family member, as well as school staff, positioning primary care as a major site for early intervention (Rowe et al., 2014). Despite this, such activity remains under-represented in the evaluation literature, which disproportionately focuses on school-based programmes when considering sites for early-intervention (Mughal et al., 2019; Nawaz et al., 2024).

Across the studies, psycho-educational programmes and brief mindfulness or expressive-therapeutic group interventions showed the most consistent promise, particularly in secondary-school and university populations (Argento et al., 2022; Baetens et al., 2020; Roberts et al., 2019a; Zare et al., 2023). While many evaluations reported some positive impact on self-harm thoughts or behaviours, effects were typically modest and often short-lived (Baetens et al., 2020; Fukumori et al., 2017; Muehlenkamp et al., 2010). Interventions in primary care more frequently targeted improvements in identification, referral and access to specialist services rather than direct reductions in self-harm behaviour, reflecting a focus on pathway facilitation over clinical change (Bailey et al., 2019; Bellairs-Walsh et al., 2020; Bennewith et al., 2002; Mughal et al., 2023).

A smaller subset of studies assessed digital delivery directed at children and young people, including app-based (Cliffe et al., 2023) and online peer-led (Cipriano et al., 2022) approaches. A further group of studies were directed at primary-care or school staff, providing training or guidance to enhance recognition and response (Birrane et al., 2015; Lloyd-Richardson et al., 2020). These resources were generally well-received and appeared to enhance engagement and confidence (Cipriano et al., 2022; Cliffe et al., 2023) but robust evidence of sustained clinical benefit from these digital approaches remains limited (Cipriano et al., 2022; Cliffe et al., 2023). Qualitative findings highlighted the value of embedding early-intervention activity within whole-school approaches, involving young people in intervention design, and ensuring clear referral pathways and adequate resourcing (Cliffe & Stallard, 2023; Lloyd-Richardson et al., 2020; Roberts et al., 2019b; Townsend et al., 2022).

Methodological limitations were common: many trials were limited by small sample sizes (Argento et al., 2022; Cipriano et al., 2022; Fukumori et al., 2017) had short follow-up (typically < 12 weeks) (Argento et al., 2022; Baetens et al., 2020; Fukumori et al., 2017; Muehlenkamp et al., 2010 *)* and most samples were drawn from high-income settings and adolescent populations, limiting generalisability across developmental stages and geographic contexts. These patterns reflect broader features of the self-harm intervention evidence base for young people (Witt et al., 2021). Safety monitoring was inconsistent: only four of the 22 included studies built systematic harm monitoring into the study design (Baetens et al., 2020; Bjärehed et al., 2013; Hart et al., 2020; Muehlenkamp et al., 2010), and a further four passively noted transient distress or operated a safety protocol without systematic measurement (Argento et al., 2022; Fukumori et al., 2017; Mughal et al., 2024; Roberts et al., 2019a). This highlights a critical gap in the evidence base and reinforces calls for all future evaluations to incorporate systematic harm-monitoring protocols from the outset (Foulkes & Andrews, 2023), ensuring that potential risks are identified and addressed before interventions are implemented widely in educational establishments (Guzman-Holst et al., 2025).

Findings indicate preliminary effectiveness for certain school-based interventions, but key evidence gaps persist around primary-care-led delivery (Moriarty et al., 2021; Mughal et al., 2019; Soneson et al., 2020), long-term outcomes (Fox et al., 2020; Witt et al., 2021) and consistent safety monitoring (Foulkes & Andrews, 2023; Lodewyk et al., 2023; NICE, 2022). Addressing these gaps will require adequately powered, multi-site trials with ≥ 12-month follow-up (Fox et al., 2020; Witt et al., 2021), robust harm-monitoring frameworks aligned to guidance (Foulkes & Andrews, 2023; NICE, 2022), and adaptation for different developmental stages, cultural contexts and service settings (NICE, 2022; NHS England, 2023).

### Comparison with wider evidence base and guidelines

The findings of this review align with and extend previous systematic reviews of self-harm interventions for children and young people across all clinical settings. Witt et al. (2021) conducted a Cochrane review of 17 RCTs evaluating interventions for self-harm in children and adolescents across all treatment settings, finding little evidence of beneficial effects for any of the therapeutic approaches examined, spanning individual, group, family and remote modalities. Importantly, most trials included in the Cochrane review were judged to carry some risk of bias, with weaknesses most commonly observed in blinding of outcome assessors, selection of reported results, and measurement of repetition of self-harm. The review identified only weak evidence for DBT adapted for adolescents, with short follow-up periods (typically less than 12 months) limiting confidence in the durability of any observed effects. Fox et al. (2020) conducted a comprehensive meta-analysis of 1,125 RCTs spanning nearly 50 years across all populations and settings, including children, revealing uniformly small intervention effects with no evidence that efficacy had improved over five decades. Harris et al. (2022), focusing exclusively on child and adolescent populations across 112 articles and 558 effect sizes, found that nearly all interventions produced nonsignificant reductions in self-injurious thoughts and behaviours, with findings largely consistent regardless of intervention type, treatment target, or sample characteristics. These parallels highlight that the limited durability of effects and the lack of consistent safety monitoring identified in the present review are not unique to educational or primary-care settings but represent systemic issues across the self-harm intervention evidence base for young people.

The high proportion of null or mixed findings in this review (8 of 12 quantitative studies) warrants critical reflection. Whilst the small to moderate effect sizes observed in the remaining four studies are consistent with the broader evidence base, they raise questions about whether existing intervention approaches are adequately targeting the mechanisms that maintain self-harm in young people. Fox et al. (2020) observation that five decades of increasing research activity have not yielded improvements in intervention efficacy suggests that refinement of existing approaches alone may be insufficient; rather, experimental research designed to identify the causal mechanisms underlying self-harm is needed so that interventions can be developed to target necessary causes rather than correlates or distal risk factors. Crucially, the pursuit of mechanistic understanding must not come at the expense of participant safety; robust harm-monitoring protocols should be integral to any experimental design testing novel or existing interventions.

### Harm monitoring and iatrogenic effects

The review's emphasis on systematic harm monitoring addresses a concern that extends beyond methodological rigour to ethical responsibility. Foulkes and Andrews (2023) propose that mental health awareness efforts, whilst enabling better recognition of genuine difficulties, may also lead to overinterpretation, whereby individuals misinterpret milder or transient distress as indicative of a mental health problem. This overinterpretation can become self-fulfilling: adopting psychiatric terminology may alter self-concept and behaviour in ways that ultimately exacerbate symptoms. The authors note that adolescents may be particularly susceptible, given their heightened proneness to rumination, peer influence, and media messaging, concerns directly relevant to the present review, in which the majority of interventions involve psycho-educational content delivered universally in school settings. Evidence from large-scale trials demonstrates that harm is not randomly distributed but disproportionately affects vulnerable subgroups. The MYRIAD trial, involving 8,376 students across 84 UK secondary schools, found no evidence that universal mindfulness training was superior to standard provision on any co-primary outcome, with the intervention arm showing marginally worse outcomes on five secondary measures including hyperactivity/inattention and teacher-reported emotional symptoms (Kuyken et al., 2022). Guzman-Holst et al. (2025) scoping review of school-based group interventions found that 10 of 112 evaluated interventions (8.93%) reported at least one negative outcome; among the subset of 15 studies rated as high quality (low risk of bias), the proportion reporting a negative outcome rose to 5 of 15 (33.3%), suggesting that more rigorous evaluation is more likely to detect harm that studies with greater risk of bias may miss. In the present review, four of the 22 included studies (18%) built systematic adverse-event monitoring into the study design (Baetens et al., 2020; Bjärehed et al., 2013; Hart et al., 2020; Muehlenkamp et al., 2010), a further four operated a safety protocol or passively noted transient distress without systematic measurement (Argento et al., 2022; Fukumori et al., 2017; Mughal et al., 2024; Roberts et al., 2019a), and 14 made no reference to adverse-event monitoring of any kind. The proportion with a described monitoring protocol (18%) is comparable to the 16% (19/117) of paediatric psychosocial intervention studies in Lodewyk et al. (2023) review that used a formal protocol to guide adverse-event monitoring, indicating that this deficit is not unique to educational and primary-care settings.

It might reasonably be argued that transient distress during engagement with emotionally difficult material represents an expected and clinically unremarkable feature of psychoeducational work, particularly among adolescents whose cognitive-emotional regulatory capacities are still developing. The concern here, then, is not that distress occurs, but that without systematic monitoring, researchers and practitioners cannot determine whether it is genuinely transient or whether it persists and escalates in a subset of participants. Harm is not randomly distributed: Guzman-Holst et al. (2025) found that negative outcomes were disproportionately concentrated in already-vulnerable subgroups. Distress that resolves for most participants may therefore mask sustained deterioration in those least equipped to manage it. That the long-term outcomes of the reviewed interventions are largely unreported makes it impossible to determine whether in-session distress prefigures or is unrelated to downstream harm, an epistemic gap that is itself an argument for systematic monitoring, not against it.

Of course, the absence of reported harm monitoring does not necessarily indicate that no monitoring occurred; some research teams may have had clinical safeguards or adverse-event procedures in place that were simply not described in the published account. This distinction matters, however, only operationally. From the standpoint of transparency and replicability, reporting harm monitoring is a methodological requirement. Without it, readers, commissioners, and practitioners cannot evaluate the safety conditions under which an intervention was tested, cannot replicate those conditions, and cannot assess whether findings generalise to settings with different safeguards. The deficit identified here is therefore a gap in the evidence base regardless of what occurred during the studies themselves.

### Alignment with national policy and guidance

It is important to distinguish between two forms of monitoring relevant to this field. NICE (2022) emphasises ongoing monitoring as integral to risk management and individualised intervention delivery, requiring practitioners to assess and reassess self-harm risk as part of clinical care for each young person. This review's findings highlight a complementary but distinct concern: the need for systematic monitoring of potential adverse effects arising from the interventions themselves, particularly when delivered universally in educational settings. Whilst NICE monitoring addresses individual clinical risk, the harm-monitoring protocols advocated here address the possibility that interventions may inadvertently exacerbate distress at a programme level, a concern substantiated by evidence from the large-scale trials discussed above (Guzman-Holst et al., 2025; Kuyken et al., 2022). Both forms of monitoring are essential, but the limited use of harm-monitoring frameworks in the evidence base represents a critical gap. At trial level, the CONSORT Harms 2022 reporting framework (Junqueira et al., 2023) provides updated guidance on adverse-event reporting in RCTs and its systematic application to future trials in educational and primary-care settings is strongly recommended. At a clinical level, NICE (2022) guidance addresses individual risk monitoring during treatment but does not specify protocols for programme-level adverse-event surveillance in universal educational interventions. The definition, measurement, and reporting of adverse events in paediatric psychosocial intervention research remains inconsistent and unstandardised, with no validated harm-monitoring framework currently established for this population (Lodewyk et al., 2023). The implications of the present review therefore include an explicit call for the development, piloting, and standardisation of such a framework, specifying minimum reporting requirements, triggers for safeguarding escalation, and procedures for detecting population-level adverse effects, within which self-harm interventions in educational and primary-care settings would represent a priority application.

Collectively, these comparisons indicate that whilst psycho-educational and brief psychotherapeutic programmes may offer some benefit, the evidence base remains insufficient to support confident widespread implementation. The high proportion of null findings, small to moderate effect sizes, and inadequate harm monitoring raise questions about whether existing approaches warrant the scale of investment currently being directed toward them. Implementation should proceed cautiously and in alignment with national guidelines, with systematic evaluation and transparent harm monitoring as essential components of any rollout.

## Conclusion

Preliminary evidence from 22 studies suggests that psycho-educational and brief psychotherapeutic programmes can reduce self-harm ideation and/or behaviour among young people, particularly in school settings, with some additional promise for integrated service-link models and targeted digital resources. However, confidence in these findings is limited by small sample sizes, short follow-up periods, methodological variability, and inconsistent monitoring for potential harms. Evidence from primary care remains scarce, despite its central role in real-world help-seeking and intervention delivery, underscoring a research bias towards educational settings. These findings are consistent with wider evidence that intervention effects for self-harm in young people remain small to nonsignificant despite decades of increasing research activity (Fox et al., 2020; Harris et al., 2022; Witt et al., 2021), and that systematic adverse-event monitoring is rarely undertaken (Lodewyk et al., 2023). A strategic research agenda is therefore required. Within primary care specifically, the evidence base is particularly limited: the present review identified only six studies, of which only one is a randomised controlled trial, the most pressing needs are: (i) adequately powered trials of brief GP consultation-based interventions for young people who self-harm, building on models of brief psychological intervention already piloted in adult primary care; (ii) evaluation of integrated GP–CAMHS liaison pathways; and (iii) feasibility and co-design studies involving young people and GPs to establish acceptable and practical formats before full-trial investment. Across both settings, future trials should incorporate ≥ 12-month follow-up, robust harm-monitoring protocols implemented before rollout at scale, and systematic approaches to ensure delivery is safe, effective, and equitable.

## Supplementary Material

Supplementary Material PRISMA_ScR_Checklist.docx

## Data Availability

Data sharing is not applicable to this article as no new data were created or analysed in this study.
